# Microbiota-immune crosstalk in livestock: implications for tick-borne disease control

**DOI:** 10.3389/fimmu.2026.1731518

**Published:** 2026-03-19

**Authors:** Miray Tonk-Rügen, Thorben Schilling, Alejandro Cabezas-Cruz, Ludwig E. Hoelzle

**Affiliations:** 1 Institute for Insect Biotechnology, Justus Liebig University of Giessen, Giessen, Germany; 2 Institute of Animal Science, Department of Livestock Infectiology and Environmental Hygiene, University of Hohenheim, Stuttgart, Germany; 3 HoLMiR-Hohenheim Center for Livestock Microbiome Research, University of Hohenheim, Stuttgart, Germany; 4 ANSES, INRAE, Ecole Nationale Vétérinaire d’Alfort, UMR BIPAR, Laboratoire de Santé Animale, Maisons-Alfort, France

**Keywords:** livestock, microbiome, natural antibody, ticks, tick-borne diseases

## Abstract

Globally, livestock health, which impacts animal welfare and agricultural productivity, is continuously threatened by tick-borne diseases (TBDs). The growing issues of acaricide overuse in livestock, emerging resistance, and vector adaptation to climate change require novel and sustainable intervention strategies. Recent advances in microbiome research reveal how host and vector microbiota influence immune responses, particularly through natural antibodies (nAbs) that modulate vector competence and pathogen transmission. In livestock, nAbs targeting microbial glycans are heritable, measurable, and linked to health outcomes. In cattle, nAb titers to bacterial antigens are associated with mastitis risk and longevity, while in pigs, early-life nAb levels are proposed as resilience markers. Studies in poultry further demonstrate the importance of high nAb phenotypes for health and production. These findings highlight nAbs as both immunological markers and potential targets for genetic selection to improve disease resistance. Emerging interventions, such as anti-microbiota vaccines and immunobiotics, aim to modulate nAb repertoires, disrupt pathogen colonization, and enhance disease resilience. Additionally, microbial glycans serve as key targets for inducing cross-reactive immunity against TBDs. Manipulation of the livestock microbiota through diet, probiotics, and prebiotics shows promise in diversifying nAb profiles and improving robustness against infection. Despite these advances, research gaps remain, particularly in establishing causality and practical feasibility in livestock systems. This review emphasizes the need for integrative research across immunology, microbiology, and veterinary sciences to leverage microbiota–immune interactions in enhancing livestock resilience against TBDs, exploring how nAbs shaped by the gut microbiota can modulate tick microbiomes and impact pathogen transmission.

## 1 Introduction

Livestock health and productivity are essential for global food security and economic stability, yet infectious diseases, especially those transmitted by ticks—remain a major challenge. Tick-borne diseases (TBDs) are a growing threat worldwide, with incidence increasing due to climate change, expanding tick distribution, and the overuse and resulting resistance to acaricides (1–7). Ticks transmit a diverse range of protozoan, bacterial, and viral pathogens to livestock (8–12). For example, the protozoa *Babesia bovis* and *Theileria parva* cause bovine babesiosis and East Coast fever, respectively, both of which lead to high mortality in cattle (13–15). These pathogens often manipulate host immune responses and evade detection, complicating treatment and prevention efforts (16–20). The burden of TBDs is substantial, with economic losses from reduced productivity, increased mortality, and disease management costs (21–25). Although live vaccines containing attenuated parasites are available, TBDs remain inadequately controlled, highlighting the urgent need for new preventive strategies to combat acute diseases and the spread of parasites into non-endemic regions (18, 26–28). Conventional tick control measures such as chemical acaricides, rotational grazing, and habitat modification face challenges such as resistance, environmental toxicity, and limited long-term efficacy (29–34). This creates a pressing need for novel, biology-based alternatives.

The microbiota — comprising diverse communities of bacteria, viruses, and fungi that reside in both hosts and vectors—has emerged as a crucial factor shaping host–pathogen–vector interactions (35–38). These microbial communities play essential roles in immune system development, metabolism, and susceptibility to infection (39, 40). In livestock, gut and other site-specific microbiota interact dynamically with the immune system to modulate defenses against vector-borne infections (41–43). In contrast, tick microbiota affects the ability of ticks to acquire and transmit pathogens, further influencing disease transmission (37, 37, 44–46).

Natural antibodies (nAbs), key components of innate immunity, are produced early in life without prior exposure to pathogens, playing crucial roles in pathogen neutralization, microbiota recognition, and cross-reactive defense in all vertebrates, including humans (47–51). They are produced by B-1 cells and provide a first-line defense by binding a broad range of exogenous and self-antigens (52–54).

**Figure 1:**
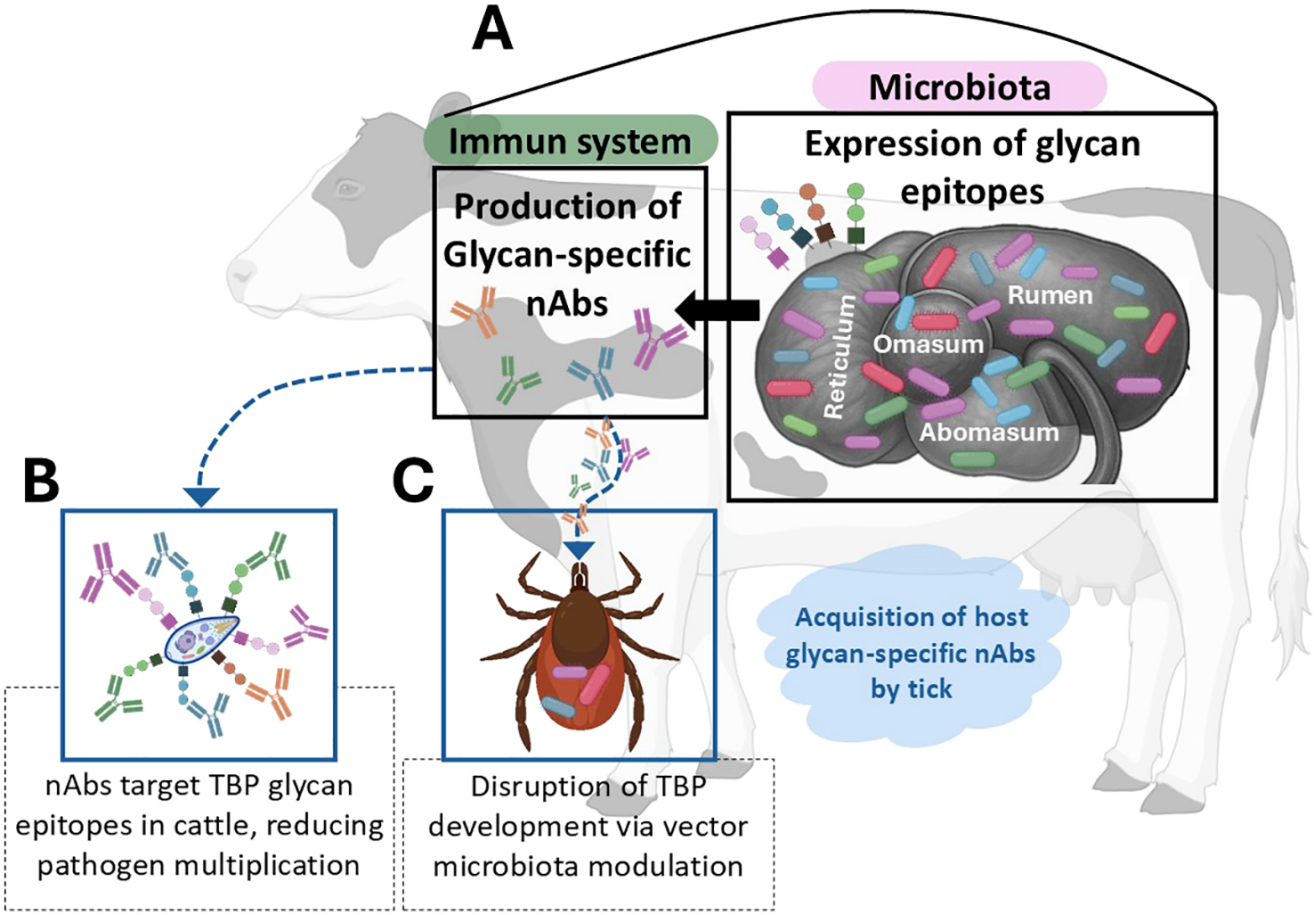
Natural Antibodies (nAbs): a dual-function immune component: **(A)** Cattle gut microbiota harbors bacteria that express glycan epitopes, thereby inducing the production of glycan-specific nAbs. **(B)** Cattle acquire tick-borne pathogen particles containing glycan epitopes through tick bites, triggering recognition by glycan-specific nAbs. These antibodies may facilitate the control or neutralization of the pathogen, reducing its capacity to multiply. Alternatively, **(C)** Ticks obtain these nAbs during the bloodmeal. These antibodies can access the tick gut lumen and target bacteria expressing glycan epitopes within the tick microbiota, resulting in alterations to the bacterial community composition and structure. nAbs-driven modulation of the tick microbiota may reduce the development of tick-borne pathogens (TBPs), thereby lowering pathogen transmission rates.

nAbs recognize conserved microbial glycans, such as —such as lipopolysaccharides (LPS), lipoteichoic acids (LTA), peptidoglycans (PGN)), as well as Keyhole Limpet Hemocyanin (KLH) and β-glucans, which are especially relevant in livestock (48, 55–57). While α-Gal is a known model epitope, livestock naturally express this glycan, making other microbial glycans more significant for nAb targeting, modulated by microbial colonization and probiotics (48, 58, 59). For examples, probiotic supplementation has been linked to increased nAb levels in chickens, enhancing both mucosal and systemic immunity (58, 60). This presents opportunities for microbiota-based interventions in livestock, with potential effects on TBD transmission by (i) neutralizing pathogens via pre-existing nAbs and (ii) altering the tick gut microbiome (56, 59). These relationships are summarized in **Figure 1**, which outlines the conceptual framework linking microbiota, nAbs, and TBP transmission.

In this review, we explore microbiota-driven immune crosstalk as a novel avenue to enhance livestock resistance against TBDs. We focus on how gut microbiota can shapes nAb production in livestock and, how these antibodies can modulate tick gut microbiome and interfere with TBPs. We also emphasize the translational potential of specific microbial epitopes and interventions. This approach will facilitate the identification of robust findings versus knowledge gaps and support the design of next-generation probiotic or vaccine strategies to reduce the burden of TBDs in livestock.

## 2 Importance of gut microbiota

The gut is a complex ecosystem of host cells, microbiota, and available nutrients (61). The microbiota constitutes a diverse ecological community encompassing commensal, symbiotic, and pathogenic microorganisms, which include bacteria, viruses, archaea, fungi, and protozoa (62–64). The gut microbiota composition is likely to affect many organ systems, including the cardiovascular, neural, immune, and metabolic systems (61, 65–70). Importantly, it plays a pivotal role in gut immune homeostasis and response (71, 72). The gut microbiota can influence the scope and quality of the immune system response; in turn, the immune system participates in regulating the localization and composition of the gut microbiota (71, 73). It has been shown that diet and nutrients significantly affect gut microbiota composition and its connection to immunological pathways (74).

### 2.1 Livestock gut microbiota and host immunity

Advances in shotgun metagenomics have revolutionized our understanding of microbial communities and their functionality (75, 76). The gut microbiota of livestock comprises diverse communities of bacteria, fungi, archaea, protozoa, plasmids, and viruses (76, 77).

The importance of the gut microbiome in livestock extends beyond digestion and nutrient absorption to maintaining immune homeostasis and pathogen defense. Extensive research in cattle, sheep, goats, and pigs demonstrates its critical roles in animal health and productivity by supporting gut development, digestion, and immune function (76, 78–87). Compared to other ruminants, studies on the cattle gut microbiome are especially comprehensive, mapping microbial communities across various regions of the gastrointestinal tract (88). Particularly in the rumen, studies have uncovered numerous novel bacterial taxa and revealed complex interactions between the microbiome, its metabolome, and the host, further highlighting the microbiota’s impact on digestion and overall health (75, 76, 83, 89).

Microbiota composition and stability in livestock are profoundly influenced by factors such as diet, environment, and antimicrobial use (90, 91). Feeding practices influence microbial diversity along the gastrointestinal tract, and parasite infections have been linked to alterations in microbiome composition (79, 92–94). A balanced microbiota maintains a symbiotic relationship with the host, modulating immune responses, preserving homeostasis, and preventing harmful inflammatory reactions against commensals, while simultaneously protecting the host from pathogenic invasion and the overgrowth of indigenous pathobionts (95, 96). Disruptions in this balance, or dysbiosis, have been associated with increased susceptibility to infections and inflammatory diseases (87). Furthermore, antibiotic-induced dysbiosis may exacerbate disease by promoting inflammatory immune responses (97, 98). Strategies to modulate the microbiota—including probiotic and prebiotic supplementation, dietary adjustments, and microbiota transplantation—have been explored to improve feed efficiency and disease resistance (76). However, the effectiveness of probiotics such as *Lactobacillus* and *Bifidobacterium*, and prebiotics such as inulin and oligosaccharides, varies across species and diets (76).

## 3 Microbiota-immune system interactions: mechanisms of natural antibody modulation

Microbiota–immune system interactions influence both innate and adaptive immunity, shaping pathogen clearance and immune memory (95, 99–102). While the detailed mechanisms by which microbiota alterations affect infection susceptibility in livestock remain under-explored, existing studies highlight their significant impact. For instance, it was highlighted the critical role of gut microbiota in modulating immune responses and influencing disease susceptibility (102).

Gut bacteria, through characteristic epitopes such as glycans, trigger the production of nAbs, which target conserved microbial glycans—ubiquitous pathogen-associated molecular patterns (PAMPs) central to host–microbe interactions (57). In dairy cattle, nAb titers are genetically associated with mastitis risk, longevity, and immune competence (103). Genome-wide association studies have identified SNPs linked to variation in nAb responses, supporting their potential as indicator traits (104). In pigs, early-life nAb levels are heritable and have been proposed as markers of resilience under polymicrobial challenge (105). High nAb levels correlate with increased fitness and pathogen resistance in multiple species: wild boar with high nAbs showed resilience to classical swine fever (106), In poultry, selection lines bred for high anti-KLH nAbs exhibited improved resistance to *E. coli* and show consistent production and health benefits (107), validating the robustness of the trait across species. These findings highlight that livestock nAbs are not only immunological features but also promising targets for genetic selection, potentially integrated into breeding programs, genomic selection, or microbiota-based strategies to enhance heritable immune traits and boost livestock resilience.

nAbs act systemically in livestock, including at skin and blood sites where ticks interact with their hosts. While the role of nAbs in shaping host–ectoparasite microbiome interactions remains underexplored, glycan-specific nAbs, including those targeting epitopes like α-Gal, can influence vector microbiomes and affect tick fitness (36, 55, 108–111). Notably, nAbs provide the first line of protection against vector-borne pathogens such as Dengue virus and *Plasmodium* spp., while also potentially targeting epitopes like α-Gal in experimental models (48, 95, 112–116). Microbial-associated molecular patterns (MAMPs)—including lipopolysaccharides, peptidoglycans, and flagellins—activate pattern recognition receptors (PRRs) on immune cells, triggering B-cell activation and differentiation into antibody-producing plasma cells (117, 118). This immune response is essential for defending against vector-borne pathogens.

Evidence also points to nAbs shaping vector microbiomes. Glycan-specific nAbs can modulate tick gut microbiota and reduce vector fitness (36, 49). Microbiota-targeted vaccination in canaries and mice reduces pathogen prevalence in mosquitoes (110) and ticks (36). For example, *Borrelia afzelii* colonization in *Ixodes ricinus* was reduced following anti-microbiota vaccination, and avian malaria transmission by mosquitoes was suppressed by immunization with microbial antigens (110). Zebrafish fed probiotics expressing α-Gal antigens developed strong nAb responses and improved resistance to mycobacterial infection (119). These findings suggest that immunobiotics—beneficial microbes or their derivatives that modulate immune responses (119)—could be used to strategically enhance nAb production in livestock.

## 4 Tick microbiota: shaping vector competence and pathogen transmission

Ticks harbor diverse microbial communities that influence their physiology, immunity, and ability to transmit pathogens (36, 120). The composition of the tick microbiota varies with species, developmental stage, environment, and host blood meal (45, 121). Core taxa include genera such as *Rickettsia*, *Coxiella*-like endosymbionts, and *Francisella*-like endosymbionts, which may contribute to tick nutrition and reproduction (45, 122). Disruption of these symbionts can impair tick development and survival, underscoring their biological relevance (122).

The role of tick microbiota in shaping vector competence is increasingly recognized. Recent studies have highlighted the potential of microbiota manipulation as a strategy to reduce pathogen prevalence in ticks (36, 37, 123). Perturbations of the tick microbiome through antibiotics or environmental factors can alter pathogen acquisition and transmission (37, 123). Dysbiosis in *Ixodes scapularis* has been associated with enhanced *Borrelia burgdorferi* colonization (37). Similarly, vaccination against specific symbionts can reduce tick fitness and pathogen prevalence (36). These findings highlight the potential of microbiota modulation as a strategy to disrupt pathogen transmission.

Host-derived antibodies can also indirectly alter tick microbiota. Glycan-specific nAbs raised by microbiota-targeted vaccines have been shown to shift the composition of the tick gut microbiome and reduce vector fitness (36). Glyco-immunogenic bacteria within the gut microbiome can stimulate the production of glycan-specific nAbs, which may directly target vector-borne pathogens, shape the vector microbiota, and trigger immune signaling pathways that influence pathogen development (109, 116).

Microbiota-targeted vaccination in canaries and mice has been shown to lower pathogen prevalence in mosquitoes and ticks (124, 125), highlighting the potential of nAbs to modulate vector microbiomes and impair vector fitness and competence. Altering the vector microbiome may also impact the vector’s immune system through the activation of key signaling pathways (126, 127).

Host–vector microbiota interactions are reciprocal. Blood meals deliver host-derived immune molecules and metabolites that shape the microbial balance inside the tick gut (45). Conversely, tick saliva introduces microbial and immunomodulatory components back into the host, influencing host immunity and microbiota composition (128–130). These bidirectional influences suggest that interventions targeting either host or vector microbiota could have cascading effects on pathogen transmission cycles.

We hypothesize that nAbs produced by livestock in response to microbiota may directly influence the tick microbiome, potentially by altering microbial communities within the tick gut. This, in turn, could impact the tick’s vector competence by either enhancing or inhibiting the colonization of tick-borne pathogens. Experimental models could test this hypothesis by examining shifts in tick microbiota composition following nAb modulation in livestock.

Although compelling results have been obtained in mosquitoes, poultry, and rodent models, extrapolation to livestock–tick systems for modulation nAb require caution. The complexity of ruminant microbiota, variation among tick species, and environmental heterogeneity mean that outcomes observed in experimental models may not directly translate to agricultural settings. More integrative and livestock-focused studies are required to test whether microbiota-based interventions can reliably disrupt TBP transmission in real-world systems. While studies in mosquitoes and zebrafish have shown microbiota–immune interactions impacting pathogen transmission, evidence in livestock–tick systems is sparse. For instance, in cattle, microbiota modulation could influence tick gut microbiomes and reduce pathogen acquisition, though this remains largely untested in real-world agricultural settings.

## 5 Strategies and practical implementation of microbiota modulation for enhanced disease resistance in livestock

**Figure 2:**
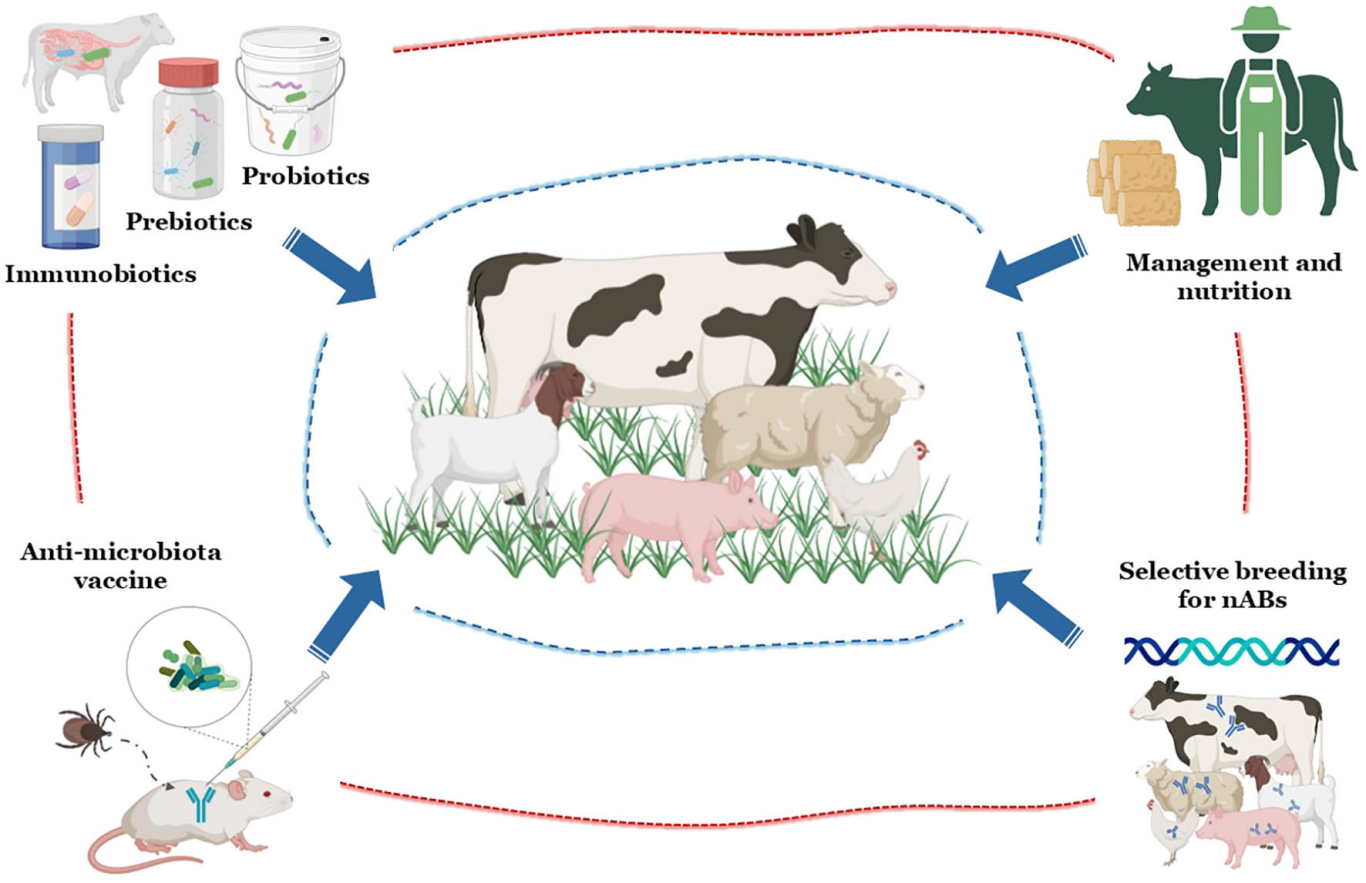
Integrated microbiota-based strategies to enhance livestock resistance to tick-borne diseases: The figure illustrates complementary approaches linking microbiota modulation and immune enhancement to reduce tick-borne pathogen transmission in livestock. Key interventions include probiotics, prebiotics, and immunobiotics to strengthen gut health and natural antibody (nAb) responses; anti-tick vaccines targeting vector microbiota; selective breeding for higher nAb titers; and improved management and nutrition to sustain immune resilience. Together, these strategies promote a sustainable, One Health-oriented framework for tick-borne disease control.

The limitations of chemical acaricides and conventional vaccines have driven interest in microbiota-based approaches to control TBPs. These strategies focus on exploiting the host–microbiota–immune axis to improve livestock resilience and reduce tick vector competence. Various avenues are being explored, including probiotics, prebiotics, postbiotics, anti-microbiota vaccines, selective breeding, management and nutrition (36, 125, 131, 132) (**Figure 2**).

Translating microbiota-based strategies into practical applications for livestock requires consideration of production environments, economic feasibility, and compatibility with existing management practices. While experimental studies demonstrate proof-of-concept, scaling to real-world systems involves several challenges and opportunities. Pilot studies in cattle, pigs, and poultry are essential to evaluate the effectiveness and scalability of these interventions.

### 5.1 Probiotics and prebiotics supplementation

As discussed earlier, probiotic supplementation can modulate host microbiota composition, enhance gut barrier function, and stimulate immune responses relevant to pathogen resistance (61, 133–135).

In poultry, probiotic administration has improved antibody responses to vaccination and reduced colonization by pathogenic bacteria such as *Salmonella* and *E. coli* while also improving growth performance (59, 136, 137). For example, multi-strain *Lactobacillus* probiotics have been shown to enhance growth and gut health, with *L. salivarius* reducing *E. coli* colonization, and Lavipan, containing *Lactococcus*, *Lactobacillus*, and *Saccharomyces*, lowering coliform levels and boosting weight gain in chicken (138–140). Prebiotic fibers, such as inulin and oligosaccharides, promote the growth of beneficial microbial taxa and their metabolites, including short-chain fatty acids, which influence B-cell metabolism and nAb production (141–145). In dairy cattle, for instance, supplementation with yeast-derived products has improved rumen function and immune responses, with some evidence linking dietary modulation to altered antibody profiles (146–149). However, the effects of probiotic and prebiotics can be strain- and species-specific, and may vary depending on host’s physiological state, highlighting the need for tailored microbiota modulation strategies in livestock health and production (150).

Although direct evidence from livestock–tick systems is lacking, these findings suggest that diet-based microbiota modulation could enhance nAb diversity and support livestock resilience against polymicrobial infections and reduce TBP transmission. These approaches could potentially enhance resilience Nonetheless, results can be inconsistent across species and production systems due to factors such as diet, host genetics, and management practices, highlighting the importance of customizing microbiota modulation strategies to specific livestock needs.

### 5.2 Immunobiotics

Immunobiotics are beneficial microorganisms or microbial products that specifically modulate the immune system to enhance disease resistance (119, 151, 152), and target immune function by stimulating natural defenses, promoting antibody production, and modulating inflammatory responses (153). Therefore, they may enhance cross-reactive immunity against TBPs by shaping the nAb repertoire, with microbial glycans in livestock (154, 155).

### 5.3 Anti-microbiota vaccines

Vaccination targeting vector microbiota or symbionts is an emerging strategy to disrupt pathogen transmission (36). In ticks, vaccination against specific gut bacteria has been shown to alter microbial composition and reduce *B. afzelii* colonization, with proof-of-concept studies demonstrating promising results (36). However, translating these findings to livestock systems will require careful validation, including the development of stable, affordable vaccine formulations compatible with existing vaccination schedules. Field trials are necessary to assess efficacy across diverse tick species and ecological conditions, as well as to evaluate the ecological impacts and long-term feasibility. Integration with existing herd vaccination programs may facilitate adoption in the future, contingent upon the demonstration of safety and cost-effectiveness.

### 5.4 Selective breeding for nAbs

nAbs could serve as valuable biomarkers for selective breeding programs aimed at enhancing immune competence and disease resistance. Integration of nAb profiling into genomic selection pipelines may offer a sustainable approach to improving livestock resilience against TBPs.

In species like pigs, chicken and cattle nAb levels have been linked to increased disease resilience (103, 105, 107), suggesting their potential for inclusion in genomic selection indices to enhance livestock robustness and reduce reliance on chemical treatments, thereby providing a sustainable, cumulative strategy.

### 5.5 Integrated approaches

Integrated approaches offer the most promising avenue for reducing the burden of TBPs. A combination of diet-based microbiota modulation, targeted immunobiotics, and selective breeding, complemented by vector-focused interventions such as anti-microbiota vaccines, may provide the most effective means of reducing the burden of TBPs (36, 125, 156, 157). These approaches align with the One Health principles, connecting livestock productivity, environmental sustainability, and reduced antimicrobial use. By modulating both host and vector microbiomes, such interventions could enhance innate immunity, decrease dependence on antibiotics and acaricides, and limit TBP transmission.

### 5.6 Management and nutrition

Diet, housing, and antimicrobial exposure influence livestock microbiota composition and thereby the nAb repertoire (158–160). For example, antimicrobial use—such as intramammary ceftiofur treatment in dairy cattle—has been shown to cause persistent changes in the gut microbiome and increased antibiotic resistance, which may constrain nAb development (161). In pigs, antibiotic administration significantly altered microbial composition and reduced production of short-chain fatty acids, including decreases in *Bifidobacterium*, *Lactobacillus*, and *Ruminococcus*—likely impacting immune-metabolite interactions relevant to nAb induction (162). In contrast, diet diversification—such as transitioning cattle from grain-fed to grass-fed systems—has been associated with distinct microbiome and resistome profiles, suggesting enriched microbiota–immune interactions (163). Practical implementation will therefore need to integrate microbiota-based strategies with broader herd management practices that promote microbial diversity.

### 5.7 Systems-level considerations

Adoption of microbiota-driven interventions must account for economic and logistical realities. Interventions must be affordable for producers, compatible with existing feeding or vaccination programs, and demonstrate measurable improvements in health and productivity. Pilot field trials in cattle, pigs, and poultry will be essential to establish feasibility. These approaches should complement, rather than replace, other control measures such as acaricide use, rotational grazing, and tick vaccines to ensure long-term sustainability.

## 6 Challenges and knowledge gaps in microbiota-immune research

Although advances in microbiota and nAb research offer promising directions, several challenges currently limit translation into livestock–tick systems. Much of the mechanistic evidence is derived from model organisms and from laboratory-maintained mosquitoes and ticks. Major microbial targets, intervention strategies, and associated immune and disease outcomes across livestock, poultry, rodent, zebrafish, and tick models are outlined (**Table 1**). While these studies have provided critical insights, their application to ruminants offers a valuable basis for species-specific research, considering differences in microbiota composition, immune ontogeny, and host–vector interactions. This highlights the urgent need for cattle- and pig-specific research that reflects the complexities of livestock production systems.

**Table 1:** Comparative summary of livestock and experimental models investigating microbiota–natural antibody (nAb) interactions.

Model	Microbial targets	Types of intervention	Observed immune & disease outcomes	References
Cattle	Glycan antigens (LPS, LTA, PGN, mannan, β-glucans); rumen taxa (*Firmicutes*, *Bacteroidetes*)	Yeast-derived supplements, mannan oligosaccharide, diet shifts, selective breeding for nAb phenotypes	Improved rumen function and milk yield; systemic Ig changes; nAb titers linked to mastitis resistance, longevity; diet/antibiotics alter microbiota & resistome	(103, 146, 147, 154)
Pigs	Glycan antigens; core gut taxa (*Bifidobacterium*, *Lactobacillus*, *Ruminococcus*)	Management/nutrition changes; reduced antibiotics; probiotic/prebiotic supplementation; selection for nAb traits	Heritable early-life nAb levels; antibiotics reduce SCFA & beneficial taxa; nAbs proposed as resilience markers	(105, 163)
Poultry (chickens, layers)	Gut bacteria expressing glycan epitopes; *Salmonella*, *E. coli*	Commercial probiotics (*Lactobacillus* spp., mixes), *in-ovo*/oral probiotics, prebiotics	Probiotics boost natural antibodies, improve vaccine responses, reduce pathogens, enhance performance; high anti-KLH nAb lines resist disease	(58, 60, 107, 138)
Rodents & other model mammals	Glycan epitopes; commensal taxa	Antibiotics, FMT, defined probiotics, anti-microbiota vaccinations	SCFAs modulate B-cell metabolism and antibody responses; antibiotics reduce diversity and immune responses; microbiota→nAb→reduced pathogens	(142, 164)
Zebrafish	α-Gal–containing microbes; probiotic strains with glycan epitopes	Probiotics/immunobiotics expressing α-Gal or glycan epitopes	α-Gal probiotics induced nAbs; zebrafish gained resistance to mycobacterial infection	(55, 119)
Ticks (vector models)	Tick gut symbionts (*Coxiella*-like, *Rickettsia*, *Francisella*-like)	Anti-microbiota vaccines, antibiotic perturbation of tick microbiome	Altering tick microbiota reduces pathogen colonization, affects tick fitness & vector competence	(36, 45, 109)

Listed are microbial targets, intervention strategies, observed immune outcomes, and supporting references.

Elucidating causal mechanisms represents a key priority for future investigations. Many existing studies are correlative in nature, providing associations between microbiota modulation, nAb diversification, and pathogen outcomes without demonstrating direct mechanisms. Carefully designed experiments—including longitudinal studies, microbiota transplantation, targeted microbial depletion, and defined probiotic supplementation in livestock—will be essential to determine whether microbiota-based interventions can reliably reduce TBP transmission.

A key challenge in advancing the field is the measurement and standardization of nAbs. Although assays for glycan-specific nAbs have been developed in cattle and pigs, their application remains inconsistent across laboratories, production systems, and environmental conditions due to the lack of standardized protocols. Addressing this challenge through harmonized protocols and validating nAb assays under diverse field conditions will be crucial for enabling reliable cross-study comparisons and the development of validated biomarkers. Standardizing these methods will not only accelerate progress in microbiota-based immune interventions but also ensure their accuracy and relevance in real-world agricultural settings, ultimately unlocking the full potential of these interventions.

The ecological complexity of livestock production presents a stimulating challenge for advancing microbiota-based strategies. Factors such as diet, housing, antimicrobial exposure, and vector ecology interact to shape both microbiota and immune responses, creating an opportunity to investigate these dynamics in real-world system. Conducting large-scale field studies that account for such heterogeneity will be essential to evaluate the feasibility, effectiveness, and robustness of targeted interventions, ultimately guiding the development of practical, resilient solutions.

At the same time, understanding and managing potential unintended consequences presents an important research opportunity. Manipulating microbiota to enhance nAbs may alter beneficial symbionts or influence opportunistic pathogens, and anti-microbiota vaccines targeting vector competence could affect tick symbioses with broader ecological impacts. These considerations highlight the value of integrating ecological evaluations with technological development, ensuring that interventions are both effective and sustainable. Finally, any new microbiota-based approaches will need to be integrated into existing livestock management frameworks. Interventions will not be applied in isolation but alongside acaricides, grazing strategies, and vaccination programs. Understanding whether microbiota-targeted tools act synergistically or antagonistically with current measures remains an unresolved challenge, yet one that will determine their practical adoption and long-term sustainability.

## 7 Future perspectives: towards innovative microbiota-based therapies against tick-borne diseases

Modulating microbiota–immune interactions to enhance livestock health and reduce the burden of TBPs is a promising approach, which requires considerable refinement before it can be translated into practice. Future progress will depend on clarifying causal mechanisms, validating interventions in real production systems, and ensuring that any new tools complement rather than replace existing control measures. A key priority is to establish mechanistic pathways linking specific microbial taxa, glycan epitopes, and nAb responses to measurable outcomes in livestock. Controlled work in species like cattle and pigs, supported by functional genomics and metabolomics, will be essential to identify microbial drivers of protective antibody repertoires.

Another area where progress is needed is the development of reliable biomarkers. Glycan-specific nAbs have shown promise as indicators of immune competence. However, standardization of assays and validation across diverse breeds and environments are critical before they can be adopted in breeding programs or herd health monitoring.

Probiotics, prebiotics, and immunobiotics hold promises for shaping the nAb repertoire, but their design should prioritize microbial epitopes relevant to livestock. Similarly, anti-microbiota vaccines need to be tailored to the ecology of livestock-associated ticks and evaluated for long-term stability and safety. One of the most urgent priorities is the field validation of microbiota-based interventions. Large-scale, longitudinal trials under farm conditions are needed to test whether microbiota-based interventions genuinely improve health, reduce pathogen prevalence, and maintain productivity. Such studies must also address economic feasibility, farmer adoption, and regulatory frameworks, since these factors will ultimately determine whether interventions reach practice.

Furthermore, One Health approach, highlights the importance of livestock microbiota in influencing not only animal health and welfare but also human health. For instance, the modulation of livestock microbiota can help reduce zoonotic disease transmission and lower the reliance on antibiotics, which in turn supports the global fight against antimicrobial resistance. Understanding these interactions provides critical insights into improving public health outcomes, as strategies designed for livestock may be adaptable to human health solutions.

Taken together, future progress will depend on integrating microbiota-based approaches into broader livestock health strategies. Rather than serving as stand-alone solutions, microbiota interventions are likely to be most effective when combined with genetic selection, nutritional management, vaccination, and conventional tick control. By situating microbiota-immune interactions within this integrated framework, the field can move towards practical, sustainable, and One Health-aligned solutions for managing TBDs in livestock.

## 8 Conclusions

TBPs remain a major threat to livestock health and productivity worldwide, and their control is becoming increasingly difficult as acaricide resistance spreads and climate change expands tick distributions. Conventional approaches, while still important, are unlikely to provide durable solutions on their own. This review highlights how the microbiota and the nAbs it elicits represent an underexplored dimension of host–vector–pathogen interactions, one that could be leveraged to improve livestock resilience.

Microbiota-targeted interventions, including probiotics, prebiotics, immunobiotics, and anti-microbiota vaccines, provide novel opportunities to shape these antibody repertoires and disrupt pathogen transmission cycles. Much of the current evidence comes from non-livestock models, and causal links between microbiota modulation, antibody diversification, and disease outcomes are not yet firmly established. Standardized methods for measuring nAbs, together with large-scale field studies in livestock systems, will be necessary to move from concept to application.

Prospectively, microbiota-based strategies should not be seen as replacements for existing practices but as components of an integrated approach to tick control and animal health. By aligning microbiota research with breeding, nutrition, management, and vaccination, the field can contribute to more sustainable livestock production. Integrating these insights into practical interventions offers a path towards reducing the burden of TBDs, improving animal welfare, and advancing One Health objectives that link agriculture, the environment, and human well-being.

## References

[1] De Rouck S İnak E Dermauw W Van Leeuwen T . A review of the molecular mechanisms of acaricide resistance in mites and ticks. Insect Biochem Mol Biol. (2023) 159:103981. doi: 10.1016/j.ibmb.2023.103981, PMID: 37391089

[2] Evans A Madder M Fourie J Halos L Kumsa B Kimbita E . Acaricide resistance status of livestock ticks from East and West Africa and *in vivo* efficacy of acaricides to control them. Int J Parasitol Drugs Drug Resist. (2024) 25:100541. doi: 10.1016/j.ijpddr.2024.100541, PMID: 38761529 PMC11133915

[3] Githaka NW Kanduma EG Bishop RP . Role of climate and other factors in determining the dynamics of tick and tick-transmitted pathogen populations and distribution in western, central and eastern Africa. CAB International (2021).

[4] Gong L Diao L Lv T Liu Y Liu J Zhang W . A comprehensive review of tick-borne disease epidemiology, clinical manifestations, pathogenesis, and prevention. Anim Zoonoses. (2025). 1:254–65. doi: 10.1016/j.azn.2025.05.004, PMID: 41833502

[5] Lejal E Marsot M Chalvet-Monfray K Cosson J-F Moutailler S Vayssier-Taussat M . A three-years assessment of *Ixodes ricinus*-borne pathogens in a French peri-urban forest. Parasites Vectors. (2019) 12:551. doi: 10.1186/s13071-019-3799-7, PMID: 31752997 PMC6873405

[6] Nuttall PA . Climate change impacts on ticks and tick-borne infections. Biologia. (2022) 77:1503–12. doi: 10.1007/s11756-021-00927-2, PMID: 41826772

[7] Obaid MK Islam N Alouffi A Khan AZ da Silva Vaz I Tanaka T . Acaricides resistance in ticks: selection, diagnosis, mechanisms, and mitigation. Front Cell Infect Microbiol. (2022) 12:941831. doi: 10.3389/fcimb.2022.941831, PMID: 35873149 PMC9299439

[8] de la Fuente J Estrada-Peña A . Ticks and tick-borne pathogens on the rise. Ticks Tick Borne Dis. (2012) 3:115–16. doi: 10.1016/j.ttbdis.2012.03.001, PMID: 22609243

[9] Jongejan F Uilenberg G . The global importance of ticks. Parasitology. (2004) 129 Suppl:S3–14. doi: 10.1017/s0031182004005967, PMID: 15938502

[10] Makwarela TG Seoraj-Pillai N Nangammbi TC . Distribution and prevalence of ticks and tick-borne pathogens at the wildlife-livestock interface in Africa: a systematic review. Vet Sci. (2025) 12:364. doi: 10.3390/vetsci12040364, PMID: 40284866 PMC12031468

[11] Sang R Onyango C Gachoya J Mabinda E Konongoi S Ofula V . Tickborne arbovirus surveillance in market livestock, Nairobi, Kenya. Emerg Infect Dis. (2006) 12:1074–80. doi: 10.3201/eid1207.060253, PMID: 16836823 PMC3291068

[12] Shi J Hu Z Deng F Shen S . Tick-borne viruses. Virol Sin. (2018) 33:21–43. doi: 10.1007/s12250-018-0019-0, PMID: 29536246 PMC5866268

[13] Almazan C Tipacamu GA Rodriguez S Mosqueda J Perez de Leon A . Immunological control of ticks and tick-borne diseases that impact cattle health and production. Front Biosci (Landmark Ed). (2018) 23:1535–51. doi: 10.2741/4659, PMID: 29293449

[14] Almazán C Scimeca RC Reichard MV Mosqueda J . Babesiosis and theileriosis in north america. Pathogens. (2022) 11:168. doi: 10.3390/pathogens11020168, PMID: 35215111 PMC8874406

[15] Olds CL Mason KL Scoles GA . *Rhipicephalus appendiculatus* ticks transmit *Theileria parva* from persistently infected cattle in the absence of detectab le parasitemia: implications for East Coast fever epidemiology. Parasites Vectors. (2018) 11:126. doi: 10.1186/s13071-018-2727-6, PMID: 29499743 PMC5834894

[16] Morrison WI . The aetiology, pathogenesis and control of theileriosis in domestic animals. Rev Sci Tech. (2015) 34:599–611. doi: 10.20506/rst.34.2.2383, PMID: 26601460

[17] Rana VS Kitsou C Dumler JS Pal U . Immune evasion strategies of major tick-transmitted bacterial pathogens. Trends Microbiol. (2023) 31:62–75. doi: 10.1016/j.tim.2022.08.002, PMID: 36055896 PMC9772108

[18] Sivakumar T Hayashida K Sugimoto C Yokoyama N . Evolution and genetic diversity of. Theileria. Infect Genet Evol. (2014) 27:250–63. doi: 10.1016/j.meegid.2014.07.013, PMID: 25102031

[19] Suarez CE Alzan HF Silva MG Rathinasamy V Poole WA Cooke BM . Unravelling the cellular and molecular pathogenesis of bovine babesiosis: is the sky the limit? Int J Parasitol. (2019) 49:183–97. doi: 10.1016/j.ijpara.2018.11.002, PMID: 30690089 PMC6988112

[20] Tajeri S Langsley G . Virulence attenuation of *Theileria annulata*-transformed macrophages. Trends Parasitol. (2025) 41:301–16. doi: 10.1016/j.pt.2025.02.007, PMID: 40057452

[21] Kasaija PD Estrada-Peña A Contreras M Kirunda H de la Fuente J . Cattle ticks and tick-borne diseases: a review of Uganda’s situation. Ticks Tick Borne Dis. (2021) 12:101756. doi: 10.1016/j.ttbdis.2021.101756, PMID: 34134062

[22] Salinas-Estrella E Amaro-Estrada I Cobaxin-Cárdenas ME Preciado de la Torre JF Rodríguez SD . Bovine Anaplasmosis: Will there ever be an almighty effective vaccine? Front Vet Sci. (2022) 9:946545. doi: 10.3389/fvets.2022.946545, PMID: 36277070 PMC9581321

[23] Singh K Kumar S Sharma AK Jacob SS RamVerma M Singh NK . Economic impact of predominant ticks and tick-borne diseases on Indian dairy production systems. Exp Parasitol. (2022) 243:108408. doi: 10.1016/j.exppara.2022.108408, PMID: 36336025

[24] Suarez CE Noh S . Emerging perspectives in the research of bovine babesiosis and anaplasmosis. Veterinary Parasitol. (2011) 180:109–25. doi: 10.1016/j.vetpar.2011.05.032, PMID: 21684084

[25] van den Heever MJJ Lombard WA Bahta YT Maré FA . The economic impact of heartwater on the South African livestock industry and the need for a new vaccine. Prev Vet Med. (2022) 203:105634. doi: 10.1016/j.prevetmed.2022.105634, PMID: 35367935

[26] De Vos AJ Bock RE . Vaccination against bovine babesiosis. Ann N Y Acad Sci. (2000) 916:540–5. doi: 10.1111/j.1749-6632.2000.tb05333.x, PMID: 11193669

[27] Florin-Christensen M Suarez CE Rodriguez AE Flores DA Schnittger L . Vaccines against bovine babesiosis: where we are now and possible roads ahead. Parasitology. (2014) 141:1563–92. doi: 10.1017/S0031182014000961, PMID: 25068315

[28] Kar PP Srivastava A . Immuno-informatics analysis to identify novel vaccine candidates and design of a multi-epitope based vaccine candidate against *Theileria* parasites. Front Immunol. (2018) 9:2213. doi: 10.3389/fimmu.2018.02213, PMID: 30374343 PMC6197074

[29] Cruz-González G Pinos-Rodríguez JM Alonso-Díaz MÁ Romero-Salas D Vicente-Martínez JG Fernández-Salas A . Efficacy of rotational grazing on the control of *Rhipicephalus microplus* infesting calves in humid tropical conditions. J Parasitol Res. (2024) 2024:7558428. doi: 10.1155/2024/7558428, PMID: 39444677 PMC11496573

[30] Dzemo WD Thekisoe O Vudriko P . Development of acaricide resistance in tick populations of cattle: A systematic review and meta-analysis. Heliyon. (2022) 8:e08718. doi: 10.1016/j.heliyon.2022.e08718, PMID: 35059516 PMC8760414

[31] Nava S Rossner MV Toffaletti JR Da Luz M Rossner MB Signorini M . Strategic control of the cattle tick *Rhipicephalus microplus* applied to rotational and silvopastoral grazing systems in subtropical areas. Parasitol Res. (2024) 123:232. doi: 10.1007/s00436-024-08256-4, PMID: 38847882

[32] Nicaretta JE Dos Santos JB Couto LFM Heller LM Cruvinel LB de Melo Júnior RD . Evaluation of rotational grazing as a control strategy for *Rhipicephalus microplus* in a tropical region. Res Vet Sci. (2020) 131:92–7. doi: 10.1016/j.rvsc.2020.04.006, PMID: 32325299

[33] Paucar-Quishpe V Pérez-Otáñez X Rodríguez-Hidalgo R Cepeda-Bastidas D Pérez-Escalante C Grijalva-Olmedo J . An economic evaluation of cattle tick acaricide-resistances and the financial losses in subtropical dairy farms of Ecuador: A farm system approach. PloS One. (2023) 18:e0287104. doi: 10.1371/journal.pone.0287104, PMID: 37384770 PMC10309988

[34] Wieren SE Braks MAH Lahr J . Effectiveness and environmental hazards of acaricides applied to large mammals for tick control. Ecol Prev Lyme borreliosis. (2016) 4:265–78. doi: 10.3920/978-90-8686-838-4_19

[35] Belkaid Y Hand TW . Role of the microbiota in immunity and inflammation. Cell. (2014) 157:121–41. doi: 10.1016/j.cell.2014.03.011, PMID: 24679531 PMC4056765

[36] Mateos-Hernández L Obregón D Wu-Chuang A Maye J Bornères J Versillé N . Anti-microbiota vaccines modulate the tick microbiome in a taxon-specific manner. Front Immunol. (2021) 12:704621. doi: 10.3389/fimmu.2021.704621, PMID: 34322135 PMC8312226

[37] Narasimhan S Rajeevan N Liu L Zhao YO Heisig J Pan J . Gut microbiota of the tick vector *Ixodes scapularis* modulate colonization of the Lyme disease spirochete. Cell Host Microbe. (2014) 15:58–71. doi: 10.1016/j.chom.2013.12.001, PMID: 24439898 PMC3905459

[38] Shreiner AB Kao JY Young VB . The gut microbiome in health and in disease. Curr Opin Gastroenterol. (2015) 31:69. doi: 10.1097/MOG.0000000000000139, PMID: 25394236 PMC4290017

[39] Belkaid Y Harrison OJ . Homeostatic immunity and the microbiota. Immunity. (2017) 46:562–76. doi: 10.1016/j.immuni.2017.04.008, PMID: 28423337 PMC5604871

[40] Round JL Mazmanian SK . The gut microbiota shapes intestinal immune responses during health and disease. Nat Rev Immunol. (2009) 9:313–23. doi: 10.1038/nri2515, PMID: 19343057 PMC4095778

[41] Chen H Liu Y Huang K Yang B Zhang Y Yu Z . Fecal microbiota dynamics and its relationship to diarrhea and health in dairy calves. J Anim Sci Biotechnol. (2022) 13:132. doi: 10.1186/s40104-022-00758-4, PMID: 36307885 PMC9616619

[42] Lima FS Oikonomou G Lima SF Bicalho MLS Ganda EK de Oliveira Filho JC . Prepartum and postpartum rumen fluid microbiomes: characterization and correlation with production traits in dairy cows. Appl Environ Microbiol. (2015) 81:1327–37. doi: 10.1128/AEM.03138-14, PMID: 25501481 PMC4309715

[43] Malmuthuge N Guan LL . Understanding host-microbial interactions in rumen: searching the best opportunity for microbiota manipulation. J Anim Sci Biotechnol. (2017) 8:8. doi: 10.1186/s40104-016-0135-3, PMID: 28116074 PMC5244612

[44] Boulanger N . . doi: 10.1016/j.pt.2025.07.009, PMID: 40780971

[45] Narasimhan S Fikrig E . Tick microbiome: the force within. Trends Parasitol. (2015) 31:315–23. doi: 10.1016/j.pt.2015.03.010, PMID: 25936226 PMC4492851

[46] Wei N Cao J Zhang H Zhou Y Zhou J . The tick microbiota dysbiosis promote tick-borne pathogen transstadial transmission in a *Babesia microti*-infected mouse model. Front Cell Infect Microbiol. (2021) 11:713466. doi: 10.3389/fcimb.2021.713466, PMID: 34414133 PMC8369883

[47] Vollmers HP Brändlein S . Natural IgM antibodies: the orphaned molecules in immune surveillance. Adv Drug Delivery Rev. (2006) 58:755–65. doi: 10.1016/j.addr.2005.08.007, PMID: 16820243

[48] Wu-Chuang A Rojas A Bernal C Cardozo F Valenzuela A Romero C . Influence of microbiota-driven natural antibodies on dengue transmission. Front Immunol. (2024) 15:1368599. doi: 10.3389/fimmu.2024.1368599, PMID: 38558802 PMC10978734

[49] Cabezas-Cruz A Hodžić A Mateos-Hernández L Contreras M de la Fuente J . Tick-human interactions: from allergic klendusity to the α-Gal syndrome. Biochem J. (2021) 478:1783–94. doi: 10.1042/BCJ20200915, PMID: 33988703

[50] Holodick NE Rodríguez-Zhurbenko N Hernández AM . Defining natural antibodies. Front Immunol. (2017) 8:872. doi: 10.3389/fimmu.2017.00872, PMID: 28798747 PMC5526850

[51] Reyneveld G Savelkoul HFJ Parmentier HK . Current understanding of natural antibodies and exploring the possibilities of modulation using veterinary models. A Review. Front Immunol. (2020) 11:2139. doi: 10.3389/fimmu.2020.02139, PMID: 33013904 PMC7511776

[52] Baumgarth N . The double life of a B-1 cell: self-reactivity selects for protective effector functions. Nat Rev Immunol. (2011) 11:34–46. doi: 10.1038/nri2901, PMID: 21151033

[53] Savage HP Baumgarth N . Characteristics of natural antibody-secreting cells. Ann N Y Acad Sci. (2015) 1362:132–42. doi: 10.1111/nyas.12799, PMID: 26104151 PMC4679694

[54] Nogueira-Martins MF Mariano M . B-1 cell participation in T-cell-mediated alloimmune response. . Immunobiology. (2010) 215:264–74. doi: 10.1016/j.imbio.2009.05.007, PMID: 19581018

[55] Pacheco I Díaz-Sánchez S Contreras M Villar M Cabezas-Cruz A Gortázar C . Probiotic bacteria with high alpha-gal content protect zebrafish against mycobacteriosis. Pharmaceutics. (2021) 14:635. doi: 10.3390/ph14070635, PMID: 34208966 PMC8308674

[56] Rossouw C Ryan FJ Lynn DJ . The role of the gut microbiota in regulating responses to vaccination: current knowledge and future directions. FEBS J. (2025) 292:1480–99. doi: 10.1111/febs.17241, PMID: 39102299 PMC11927049

[57] Zerna G Cameron TC Toet H Spithill TW Beddoe T . Bovine natural antibody relationships to specific antibodies and *Fasciola hepatica* burdens after experimental infection and vaccination with glutathione S-transferase. Veterinary Sci. (2022) 9:58. doi: 10.3390/vetsci9020058, PMID: 35202313 PMC8876122

[58] Haghighi HR Gong J Gyles CL Hayes MA Zhou H Sanei B . Probiotics stimulate production of natural antibodies in chickens. Clin Vaccine Immunol. (2006) 13:975–80. doi: 10.1128/CVI.00161-06, PMID: 16960107 PMC1563569

[59] Idowu PA Mpofu TJ Magoro AM Modiba MC Nephawe KA Mtileni B . Impact of probiotics on chicken gut microbiota, immunity, behavior, and productive performance—a systematic review. Front Anim Sci. (2025) 6:1562527. doi: 10.3389/fanim.2025.1562527, PMID: 41822879

[60] Alizadeh M Bavananthasivam J Shojadoost B Astill J Taha-Abdelaziz K Alqazlan N . In ovo and oral administration of probiotic lactobacilli modulate cell- and antibody-mediated immune responses in newly hatched chicks. Front Immunol. (2021) 12:664387. doi: 10.3389/fimmu.2021.664387, PMID: 33912191 PMC8072127

[61] Azad MAK Sarker M Li T Yin J . Probiotic species in the modulation of gut microbiota: An overview. BioMed Res Int. (2018) 2018:9478630. doi: 10.1155/2018/9478630, PMID: 29854813 PMC5964481

[62] Hou K Wu Z-X Chen X-Y Wang J-Q Zhang D Xiao C . Microbiota in health and diseases. Sig Transduct Target Ther. (2022) 7:135. doi: 10.1038/s41392-022-00974-4, PMID: 35461318 PMC9034083

[63] Parfrey LW Walters WA Knight R . microbial eukaryotes in the human microbiome: ecology, evolution, and future directions. Front Microbiol. (2011) 2:153. doi: 10.3389/fmicb.2011.00153, PMID: 21808637 PMC3135866

[64] Van Hul M Cani PD Petitfils C De Vos WM Tilg H El-Omar EM . What defines a healthy gut microbiome? Gut. (2024) 73:e333378. doi: 10.1136/gutjnl-2024-333378, PMID: 39322314 PMC11503168

[65] Ma P-J Wang M-M Wang Y . Gut microbiota: A new insight into lung diseases. BioMed Pharmacother. (2022) 155:113810. doi: 10.1016/j.biopha.2022.113810, PMID: 36271581

[66] Qiu P Ishimoto T Fu L Zhang J Zhang Z Liu Y . The gut microbiota in inflammatory bowel disease. Front Cell Infect Microbiol. (2022) 12:733992. doi: 10.3389/fcimb.2022.733992, PMID: 35273921 PMC8902753

[67] Ronen D Rokach Y Abedat S Qadan A Daana S Amir O . Human gut microbiota in cardiovascular disease. Compr Physiol. (2024) 14:5449–90. doi: 10.1002/cphy.c230012, PMID: 39109979

[68] Roy Sarkar S Banerjee S . Gut microbiota in neurodegenerative disorders. J Neuroimmunol. (2019) 328:98–104. doi: 10.1016/j.jneuroim.2019.01.004, PMID: 30658292

[69] Sittipo P Lobionda S Lee YK Maynard CL . Intestinal microbiota and the immune system in metabolic diseases. J Microbiol. (2018) 56:154–62. doi: 10.1007/s12275-018-7548-y, PMID: 29492872

[70] Kamada N Seo S-U Chen GY Núñez G . Role of the gut microbiota in immunity and inflammatory disease. Nat Rev Immunol. (2013) 13:321–35. doi: 10.1038/nri3430, PMID: 23618829

[71] Gao J Xu K Liu H Liu G Bai M Peng C . Impact of the gut microbiota on intestinal immunity mediated by tryptophan metabolism. Front Cell Infect Microbiol. (2018) 8:13. doi: 10.3389/fcimb.2018.00013, PMID: 29468141 PMC5808205

[72] Wang J Hou Y Mu L Yang M Ai X . Gut microbiota contributes to the intestinal and extraintestinal immune homeostasis by balancing Th17/Treg cells. Int Immunopharmacol. (2024) 143:113570. doi: 10.1016/j.intimp.2024.113570, PMID: 39547012

[73] Nicholson JK Wilson ID . Opinion: understanding “global” systems biology: metabonomics and the continuum of metabolism. Nat Rev Drug Discov. (2003) 2:668–76. doi: 10.1038/nrd1157, PMID: 12904817

[74] Thorburn AN Macia L Mackay CR . Diet, metabolites, and “western-lifestyle” inflammatory diseases. Immunity. (2014) 40:833–42. doi: 10.1016/j.immuni.2014.05.014, PMID: 24950203

[75] Stewart RD Auffret MD Warr A Walker AW Roehe R Watson M . Compendium of 4,941 rumen metagenome-assembled genomes for rumen microbiome biology and enzyme discovery. Nat Biotechnol. (2019) 37:953–61. doi: 10.1038/s41587-019-0202-3, PMID: 31375809 PMC6785717

[76] Tardiolo G La Fauci D Riggio V Daghio M Di Salvo E Zumbo A . Gut microbiota of ruminants and monogastric livestock: an overview. Animals. (2025) 15:758. doi: 10.3390/ani15050758, PMID: 40076043 PMC11899476

[77] Claesson MJ Jeffery IB Conde S Power SE O’Connor EM Cusack S . Gut microbiota composition correlates with diet and health in the elderly. Nature. (2012) 488:178–84. doi: 10.1038/nature11319, PMID: 22797518

[78] Asanuma N Yokoyama S Hino T . Effects of nitrate addition to a diet on fermentation and microbial populations in the rumen of goats, with special reference to *Selenomonas ruminantium* having the ability to reduce nitrate and nitrite. Anim Sci J. (2015) 86:378–84. doi: 10.1111/asj.12307, PMID: 25439583

[79] Cui X Wang Z Guo P Li F Chang S Yan T . Shift of feeding strategies from grazing to different forage feeds reshapes the rumen microbiota to improve the ability of Tibetan sheep (*Ovis aries*) to adapt to the cold season. Microbiol Spectr. (2023) 11:e0281622. doi: 10.1128/spectrum.02816-22, PMID: 36809032 PMC10100778

[80] Dou S Gadonna-Widehem P Rome V Hamoudi D Rhazi L Lakhal L . characterisation of early-life fecal microbiota in susceptible and healthy pigs to post-weaning diarrhoea. PloS One. (2017) 12:e0169851. doi: 10.1371/journal.pone.0169851, PMID: 28072880 PMC5225014

[81] Ericsson AC Johnson PJ Lopes MA Perry SC Lanter HR . A Microbiological map of the healthy equine gastrointestinal tract. PloS One. (2016) 11:e0166523. doi: 10.1371/journal.pone.0166523, PMID: 27846295 PMC5112786

[82] Li A Kiani FA Liao J Liu F Chang Y-F . Editorial: The role of gut microbiota in animal gastrointestinal diseases. Front Cell Infect Microbiol. (2025) 15:1554277. doi: 10.3389/fcimb.2025.1554277, PMID: 39944721 PMC11814163

[83] Rawal S Kaur H Bhathan S Mittal D Kaur G Ali SA . Ruminant gut microbiota: interplay, implications, and innovations for sustainable livestock production. Sustain Agric Reviews: Anim Biotechnol Livestock Production 4. (2024) p:205–28. doi: 10.1007/978-3-031-54372-2_7, PMID: 41826772

[84] Sutera AM Arfuso F Tardiolo G Riggio V Fazio F Aiese Cigliano R . Effect of a co-feed liquid whey-integrated diet on crossbred pigs’ Fecal microbiota. Anim (Basel). (2023) 13:1750. doi: 10.3390/ani13111750, PMID: 37889679 PMC10252047

[85] Tardiolo G Romeo O Zumbo A Di Marsico M Sutera AM Cigliano RA . Characterization of the nero siciliano pig fecal microbiota after a liquid whey-supplemented diet. Anim (Basel). (2023) 13:642. doi: 10.3390/ani13040642, PMID: 36830429 PMC9951753

[86] Zhang K He C Wang L Suo L Guo M Guo J . Compendium of 5810 genomes of sheep and goat gut microbiomes provides new insights into the glycan and mucin utilization. Microbiome. (2024) 12:104. doi: 10.1186/s40168-024-01806-z, PMID: 38845047 PMC11155115

[87] Khalil A Batool A Arif S . Healthy cattle microbiome and dysbiosis in diseased phenotypes. Ruminants. (2022) 2:134–56. doi: 10.3390/ruminants2010009, PMID: 41725453

[88] Scicutella F Cucu MA Mannelli F Pastorelli R Daghio M Paoli P . Rumen microbial community and milk quality in Holstein lactating cows fed olive oil pomace as part in a sustainable feeding strategy. Animal. (2023) 17:100815. doi: 10.1016/j.animal.2023.100815, PMID: 37167820

[89] Wylezich C Belka A Hanke D Beer M Blome S Höper D . Metagenomics for broad and improved parasite detection: a proof-of-concept study using swine faecal samples. Int J Parasitol. (2019) 49:769–77. doi: 10.1016/j.ijpara.2019.04.007, PMID: 31361998

[90] Lin L Lai Z Zhang J Zhu W Mao S . The gastrointestinal microbiome in dairy cattle is constrained by the deterministic driver of the region and the modified effect of diet. Microbiome. (2023) 11:10. doi: 10.1186/s40168-022-01453-2, PMID: 36670455 PMC9863278

[91] O’Hara E Neves ALA Song Y Guan LL . The role of the gut microbiome in cattle production and health: Driver or Passenger? Annu Rev Anim Biosci. (2020) 8:199–220. doi: 10.1146/annurev-animal-021419-083952, PMID: 32069435

[92] Cortés A Wills J Su X Hewitt RE Robertson J Scotti R . Infection with the sheep gastrointestinal nematode *Teladorsagia circumcincta* increases luminal pathobionts. Microbiome. (2020) 8:60. doi: 10.1186/s40168-020-00818-9, PMID: 32354347 PMC7193420

[93] Wang J Fan H Han Y Zhao J Zhou Z . Characterization of the microbial communities along the gastrointestinal tract of sheep by 454 pyrosequencing analysis. Asian-Australas J Anim Sci. (2017) 30:100–10. doi: 10.5713/ajas.16.0166, PMID: 27383798 PMC5205584

[94] Wang X Hu L Liu H Xu T Zhao N Zhang X . Characterization of the bacterial microbiota across the different intestinal segments of the Qinghai semi-fine wool sheep on the Qinghai-Tibetan Plateau. Anim Biosci. (2021) 34:1921–9. doi: 10.5713/ab.20.0809, PMID: 34237935 PMC8563230

[95] Bello-Gil D Audebert C Olivera-Ardid S Pérez-Cruz M Even G Khasbiullina N . The formation of glycan-specific natural antibodies repertoire in galt-ko mice is determined by gut microbiota. Front Immunol. (2019) 10:342. doi: 10.3389/fimmu.2019.00342, PMID: 30891034 PMC6411795

[96] Dzutsev A Goldszmid RS Viaud S Zitvogel L Trinchieri G . The role of the microbiota in inflammation, carcinogenesis, and cancer therapy. Eur J Immunol. (2015) 45:17–31. doi: 10.1002/eji.201444972, PMID: 25328099

[97] Britton RA Young VB . Role of the intestinal microbiota in resistance to colonization by. Clostridium difficile. Gastroenterol. (2014) 146:1547–53. doi: 10.1053/j.gastro.2014.01.059, PMID: 24503131 PMC3995857

[98] Tong J Ma W Yang R Wang T Chen X Zhang X . Dysbiosis of the gut microbiota maybe exacerbate orf pathology by promoting inflammatory immune responses. Vet Microbiol. (2020) 251:108884. doi: 10.1016/j.vetmic.2020.108884, PMID: 33086176

[99] Heidari M Maleki Vareki S Yaghobi R Karimi MH . Microbiota activation and regulation of adaptive immunity. Front Immunol. (2024) 15:1429436. doi: 10.3389/fimmu.2024.1429436, PMID: 39445008 PMC11496076

[100] Yoo JY Groer M Dutra SVO Sarkar A McSkimming DI . Gut microbiota and immune system interactions. Microorganisms. (2020) 8:1587. doi: 10.3390/microorganisms8101587, PMID: 33076307 PMC7602490

[101] Zheng D Liwinski T Elinav E . Interaction between microbiota and immunity in health and disease. Cell Res. (2020) 30:492–506. doi: 10.1038/s41422-020-0332-7, PMID: 32433595 PMC7264227

[102] Zhang J Holdorf AD Walhout AJ . C. elegans and its bacterial diet as a model for systems-level understanding of host-microbiota interactions. Curr Opin Biotechnol. (2017) 46:74–80. doi: 10.1016/j.copbio.2017.01.008, PMID: 28189107 PMC5544573

[103] Thompson-Crispi KA Miglior F Mallard BA . Genetic parameters for natural antibodies and associations with specific antibody and mastitis in Canadian Holsteins. J Dairy Sci. (2013) 96:3965–72. doi: 10.3168/jds.2012-5919, PMID: 23587396

[104] Cordero-Solorzano J Parmentier HK Arts JAJ van der Poel J de Koning DJ Bovenhuis H . Genome-wide association study identifies loci influencing natural antibody titers in milk of Dutch Holstein-Friesian cattle. J Dairy Sci. (2019) 102:11092–103. doi: 10.3168/jds.2019-16627, PMID: 31548067

[105] Chen Y Tibbs-Cortes LE Ashley C Putz AM Lim K-S Dyck MK . The genetic basis of natural antibody titers of young healthy pigs and relationships with disease resilience. BMC Genomics. (2020) 21:648. doi: 10.1186/s12864-020-06994-0, PMID: 32962629 PMC7510148

[106] Rossi S Doucelin A Le Potier M-F Eraud C Gilot-Fromont E . Innate immunity correlates with host fitness in wild boar (*Sus scrofa*) exposed to classical swine fever. PloS One. (2013) 8:e79706. doi: 10.1371/journal.pone.0079706, PMID: 24260286 PMC3832544

[107] Berghof TVL Matthijs MGR Arts J a. J Bovenhuis H Dwars RM van der Poel JJ . Selective breeding for high natural antibody level increases resistance to avian pathogenic *Escherichia coli* (APEC) in chickens. Dev Comp Immunol. (2019) 93:45–57. doi: 10.1016/j.dci.2018.12.007, PMID: 30579935

[108] Arango J Wolc A Owen J Weston K Fulton JE . Genetic variation in natural and induced antibody responses in layer chickens. Animals. (2024) 14:1623. doi: 10.3390/ani14111623, PMID: 38891669 PMC11171384

[109] Maitre A Wu-Chuang A Aželytė J Palinauskas V Mateos-Hernández L Obregon D . Vector microbiota manipulation by host antibodies: the forgotten strategy to develop transmission-blocking vaccines. Parasites Vectors. (2022) 15:4. doi: 10.1186/s13071-021-05122-5, PMID: 34983601 PMC8725291

[110] Palinauskas V Mateos-Hernandez L Wu-Chuang A de la Fuente J Aželytė J Obregon D . Exploring the ecological implications of microbiota diversity in birds: natural barriers against avian malaria. Front Immunol. (2022) 13:807682. doi: 10.3389/fimmu.2022.807682, PMID: 35250978 PMC8891477

[111] Sinyakov MS Dror M Zhevelev HM Margel S Avtalion RR . Natural antibodies and their significance in active immunization and protection against a defined pathogen in fish. Vaccine. (2002) 20:3668–74. doi: 10.1016/s0264-410x(02)00379-1, PMID: 12399194

[112] Erturk-Hasdemir D Kasper DL . Resident commensals shaping immunity. Curr Opin Immunol. (2013) 25:450–5. doi: 10.1016/j.coi.2013.06.001, PMID: 23830047 PMC3775925

[113] Springer GF Horton RE . Blood group isoantibody stimulation in man by feeding blood group-active bacteria. J Clin Invest. (1969) 48:1280–91. doi: 10.1172/JCI106094, PMID: 4893685 PMC322351

[114] Khasbiullina NR Bovin NV . Hypotheses of the origin of natural antibodies: a glycobiologist’s opinion. Biochem (Mosc). (2015) 80:820–35. doi: 10.1134/S0006297915070032, PMID: 26541997

[115] Wijga S Bovenhuis H Bastiaansen JWM van Arendonk J a. M Ploegaert TCW Tijhaar E . Genetic parameters for natural antibody isotype titers in milk of Dutch Holstein-Friesians. Anim Genet. (2013) 44:485–92. doi: 10.1111/age.12038, PMID: 23496254

[116] Yilmaz B Portugal S Tran TM Gozzelino R Ramos S Gomes J . Gut microbiota elicits a protective immune response against malaria transmission. Cell. (2014) 159:1277–89. doi: 10.1016/j.cell.2014.10.053, PMID: 25480293 PMC4261137

[117] Chatterjee N Perrimon N . What fuels the fly: Energy metabolism in Drosophila and its application to the study of obesity and diabetes. Sci Adv. (2021) 7:eabg4336. doi: 10.1126/sciadv.abg4336, PMID: 34108216 PMC8189582

[118] Li D Wu M . Pattern recognition receptors in health and diseases. Sig Transduct Target Ther. (2021) 6:291. doi: 10.1038/s41392-021-00687-0, PMID: 34344870 PMC8333067

[119] Pacheco I Contreras M Villar M Risalde MA Alberdi P Cabezas-Cruz A . Vaccination with alpha-gal protects against mycobacterial infection in the zebrafish model of tuberculosis. Vaccines (Basel). (2020) 8:195. doi: 10.3390/vaccines8020195, PMID: 32344637 PMC7348772

[120] Bonnet SI Pollet T . Update on the intricate tango between tick microbiomes and tick-borne pathogens. Parasite Immunol. (2021) 43:e12813. doi: 10.1111/pim.12813, PMID: 33314216

[121] Andreotti R Pérez de León AA Dowd SE Guerrero FD Bendele KG Scoles GA . Assessment of bacterial diversity in the cattle tick *Rhipicephalus (Boophilus)* microplus through tag-encoded pyrosequencing. BMC Microbiol. (2011) 11:6. doi: 10.1186/1471-2180-11-6, PMID: 21211038 PMC3025832

[122] Duron O Morel O Noël V Buysse M Binetruy F Lancelot R . Tick-bacteria mutualism depends on B vitamin synthesis pathways. Curr Biol. (2018) 28:1896–1902.e5. doi: 10.1016/j.cub.2018.04.038, PMID: 29861133

[123] Adegoke A Kumar D Bobo C Rashid MI Durrani AZ Sajid MS . Tick-borne pathogens shape the native microbiome within tick vectors. Microorganisms. (2020) 8:1299. doi: 10.3390/microorganisms8091299, PMID: 32854447 PMC7563471

[124] Aželytė J Wu-Chuang A Maitre A Žiegytė R mateos-hernández L obregón D . Avian malaria parasites modulate gut microbiome assembly in canaries. Microorganisms. (2023) 11:563. doi: 10.3390/microorganisms11030563, PMID: 36985137 PMC10056159

[125] Wu-Chuang A Mateos-Hernandez L Maitre A Rego ROM Šíma R Porcelli S . Microbiota perturbation by anti-microbiota vaccine reduces the colonization of. Borrelia afzelii Ixodes ricinus. Microbiome. (2023) 11:151. doi: 10.1186/s40168-023-01599-7, PMID: 37482606 PMC10364381

[126] Estrada-Peña A Cabezas-Cruz A Obregón D . Resistance of tick gut microbiome to anti-tick vaccines, pathogen infection and antimicrobial peptides. Pathogens. (2020) 9:309. doi: 10.3390/pathogens9040309, PMID: 32331444 PMC7238099

[127] Fogaça AC Sousa G Pavanelo DB Esteves E Martins LA Urbanová V . tick immune system: what is known, the interconnections, the gaps, and the challenges. Front Immunol. (2021) 12:628054. doi: 10.3389/fimmu.2021.628054, PMID: 33737931 PMC7962413

[128] Bonnet S Kazimírová M Richardson J Šimo L . Chapter 5 - Tick saliva and its role in pathogen transmission. In: Boulanger N , editor. Skin and Arthropod Vectors. Wien Klin Wochenschr. (2018). p. 121–91. doi: 10.1016/B978-0-12-811436-0.00005-8, PMID:

[129] Kazimirova M Stibraniova I . Tick salivary compounds: their role in modulation of host defences and pathogen transmission. Front Cell Infect Microbiol. (2013) 3:43. doi: 10.3389/fcimb.2013.00043, PMID: 23971008 PMC3747359

[130] Kitsou C Fikrig E Pal U . Tick host immunity: vector immunomodulation and acquired tick resistance. Trends Immunol. (2021) 42:554–74. doi: 10.1016/j.it.2021.05.005, PMID: 34074602 PMC10089699

[131] Smolinska S Popescu F-D Zemelka-wiacek M . A Review of the influence of prebiotics, probiotics, synbiotics, and postbiotics on the human gut microbiome and intestinal integrity. J Clin Med. (2025) 14:3673. doi: 10.3390/jcm14113673, PMID: 40507435 PMC12156228

[132] Zilber-Rosenberg I Rosenberg E . Role of microorganisms in the evolution of animals and plants: the hologenome theory of evolution. FEMS Microbiol Rev. (2008) 32:723–35. doi: 10.1111/j.1574-6976.2008.00123.x, PMID: 18549407

[133] Kogut MH Swaggerty CL . Effects of prebiotics and probiotics on the host immune response. In: Callaway TR Ricke SC , editors. Direct-Fed Microbials and Prebiotics for Animals: Science and Mechanisms of Action. Spring Nature (2012). p. 61–72. doi: 10.1007/978-1-4614-1311-0_5, PMID:

[134] La Fata G Weber P Mohajeri MH . Probiotics and the gut immune system: indirect regulation. Probiotics Antimicrob Proteins. (2018) 10:11–21. doi: 10.1007/s12602-017-9322-6, PMID: 28861741 PMC5801397

[135] Zhou P Chen C Patil S Dong S . Unveiling the therapeutic symphony of probiotics, prebiotics, and postbiotics in gut-immune harmony. Front Nutr. (2024) 11:1355542. doi: 10.3389/fnut.2024.1355542, PMID: 38389798 PMC10881654

[136] Ayana GU Kamutambuko R . Probiotics in disease management for sustainable poultry production. Advanced Gut Microbiome Res. (2024) 4326438. doi: 10.1155/2024/4326438, PMID: 41641447

[137] Naeem M Bourassa D . Probiotics in poultry: unlocking productivity through microbiome modulation and gut health. Microorganisms. (2025) 13:257. doi: 10.3390/microorganisms13020257, PMID: 40005624 PMC11857632

[138] Ding S Wang Y Yan W Li A Jiang H Fang J . Effects of *Lactobacillus plantarum* 15–1 and fructooligosaccharides on the response of broilers to pathogenic *Escherichia coli* O78 challenge. PloS One. (2019) 14:e0212079. doi: 10.1371/journal.pone.0212079, PMID: 31194771 PMC6563962

[139] Reuben RC Sarkar SL Ibnat H Roy PC Jahid IK . Novel mono- and multi-strain probiotics supplementation modulates growth, intestinal microflora composition and haemato-biochemical parameters in broiler chickens. Vet Med Sci. (2022) 8:668–80. doi: 10.1002/vms3.709, PMID: 35014219 PMC8959300

[140] Smialek M Burchardt S Koncicki A . The influence of probiotic supplementation in broiler chickens on population and carcass contamination with *Campylobacter* spp. - Field study. Res Veterinary Sci. (2018) 118:312–6. doi: 10.1016/j.rvsc.2018.03.009, PMID: 29567598

[141] Davani-Davari D Negahdaripour M Karimzadeh I Seifan M Mohkam M Masoumi SJ . Prebiotics: definition, types, sources, mechanisms, and clinical applications. Foods. (2019) 8:92. doi: 10.3390/foods8030092, PMID: 30857316 PMC6463098

[142] Kim M Qie Y Park J Kim CH . Gut microbial metabolites fuel host antibody responses. Cell Host Microbe. (2016) 20:202–14. doi: 10.1016/j.chom.2016.07.001, PMID: 27476413 PMC4982788

[143] Krautkramer KA Fan J Bäckhed F . Gut microbial metabolites as multi-kingdom intermediates. Nat Rev Microbiol. (2021) 19:77–94. doi: 10.1038/s41579-020-0438-4, PMID: 32968241

[144] Liu J Tan Y Cheng H Zhang D Feng W Peng C . Functions of gut microbiota metabolites, current status and future perspectives. Aging Dis. (2022) 13:1106–26. doi: 10.14336/AD.2022.0104, PMID: 35855347 PMC9286904

[145] Yoo S Jung S-C Kwak K Kim J-S . The Role of prebiotics in modulating gut microbiota: implications for human health. Int J Mol Sci. (2024) 25:4834. doi: 10.3390/ijms25094834, PMID: 38732060 PMC11084426

[146] Alugongo GM Xiao JX Chung YH Dong SZ Li SL Yoon I . Effects of *Saccharomyces cerevisiae* fermentation products on dairy calves: Performance and health. J Dairy Sci. (2017) 100:1189–99. doi: 10.3168/jds.2016-11399, PMID: 28012624

[147] Grossi S Dell’Anno M Rossi L Compiani R Sgoifo Rossi CA . supplementation of live yeast, mannan oligosaccharide, and organic selenium during the adaptation phase of newly arrived beef cattle: effects on health status, immune functionality, and growth performance. Antibiotics. (2021) 10:1114. doi: 10.3390/antibiotics10091114, PMID: 34572696 PMC8470399

[148] Klopp RN Yoon I Eicher S Boerman JP . Effects of feeding *Saccharomyces cerevisiae* fermentation products on the health of Holstein dairy calves following a lipopolysaccharide challenge. J Dairy Sci. (2022) 105:1462–74. doi: 10.3168/jds.2021-21149, PMID: 34802742

[149] Sivinski SE Meier KE Mamedova LK Saylor BA Shaffer JE Sauls-Hiesterman JA . Effect of *Saccharomyces cerevisiae* fermentation product on oxidative status, inflammation, and immune response in transition dairy cattle. J Dairy Sci. (2022) 105:8850–65. doi: 10.3168/jds.2022-21998, PMID: 36153156

[150] Uyeno Y Shigemori S Shimosato T . Effect of probiotics/prebiotics on cattle health and productivity. Microbes Environ. (2015) 30:126–32. doi: 10.1264/jsme2.ME14176, PMID: 26004794 PMC4462921

[151] Villena J Kitazawa H . Editorial: Immunobiotics—interactions of beneficial microbes with the immune system. Front Immunol. (2017) 8:1580. doi: 10.3389/fimmu.2017.01580, PMID: 29250061 PMC5715392

[152] Villena J Aso H Rutten VPMG Takahashi H van Eden W Kitazawa H . Immunobiotics for the bovine host: their interaction with intestinal epithelial cells and their effect on antiviral immunity. Front Immunol. (2018) 9:326. doi: 10.3389/fimmu.2018.00326, PMID: 29599767 PMC5863502

[153] Koshtemirova M Sobirjonova M . Immunobiotics: novel approaches to strengthening the immune system using microorganisms. Am J BioMed Sci Pharm Innov. (2025) 5:77–9. doi: 10.37547/ajbspi/Volume05Issue10-15

[154] Ploegaert TCW Tijhaar E Lam TJGM . Natural antibodies in bovine milk and blood plasma: variability among cows, repeatability within cows, and relation between milk and plasma titers. Vet Immunol Immunopathol. (2011) 144:88–94. doi: 10.1016/j.vetimm.2011.07.008, PMID: 21839523

[155] Altena V . Biomarkers and mechanisms of natural disease resistance in dairy cows. Wageningen: Wageningen University (2016). doi: 10.18174/380150, PMID:

[156] de la Fuente J Sobrino I Villar M . Design and evaluation of vaccines for the control of the etiological agent of East Coast fever. Parasites Vectors. (2024) 17:479. doi: 10.1186/s13071-024-06517-w, PMID: 39567980 PMC11580188

[157] Ibeagha-Awemu EM Omonijo FA Piché LC Vincent AT . Alternatives to antibiotics for sustainable livestock production in the context of the One Health approach: tackling a common foe. Front Vet Sci. (2025) 12:1605215. doi: 10.3389/fvets.2025.1605215, PMID: 40881639 PMC12381954

[158] Aruwa CE Pillay C Nyaga MM Sabiu S . Poultry gut health – microbiome functions, environmental impacts, microbiome engineering and advancements in characterization technologies. J Anim Sci Biotechnol. (2021) 12:119. doi: 10.1186/s40104-021-00640-9, PMID: 34857055 PMC8638651

[159] Chen R-A Wu W-K Panyod S Liu P-Y Chuang H-L Chen Y-H . Dietary exposure to antibiotic residues facilitates metabolic disorder by altering the gut microbiota and bile acid composition. mSystems. (2022) 7:e00172–22. doi: 10.1128/msystems.00172-22, PMID: 35670534 PMC9239188

[160] Curtis-Joseph N Peterson R Brown CE Beekman C Belenky P . Mouse diet and vendor impact microbiome perturbation and recovery from early-life pulses of amoxicillin. Front Microbiomes. (2024) 3:1432202. doi: 10.3389/frmbi.2024.1432202, PMID: 41853521 PMC12993551

[161] Vasco KA Carbonell S Sloup RE Bowcutt B Colwell RR Graubics K . Persistent effects of intramammary ceftiofur treatment on the gut microbiome and antibiotic resistance in dairy cattle. Anim Microbiome. (2023) 5:56. doi: 10.1186/s42523-023-00274-4, PMID: 37946266 PMC10636827

[162] Zeineldin M Aldridge B Lowe J . Antimicrobial effects on swine gastrointestinal microbiota and their accompanying antibiotic resistome. Front Microbiol. (2019) 10:1035. doi: 10.3389/fmicb.2019.01035, PMID: 31156580 PMC6530630

[163] Zhou Y . Metagenomic analysis revealed significant changes in cattle rectum microbiome and antimicrobial resistome under fescue toxicosis. Biology. (2025). 14:1197. doi: 10.3390/biology14091197, PMID: 41007342 PMC12466995

[164] Arpaia N Campbell C Fan X Dikiy S van der Veeken J deRoos P . Metabolites produced by commensal bacteria promote peripheral regulatory T-cell generation. Nature. (2013) 504:451–5. doi: 10.1038/nature12726, PMID: 24226773 PMC3869884

